# *QuickStats: *Percentage Distribution of Deaths Involving Injuries from Recreational and Nonrecreational Use of Watercraft,* by Month — United States, 2018–2020

**DOI:** 10.15585/mmwr.mm7121a5

**Published:** 2022-05-27

**Figure:**
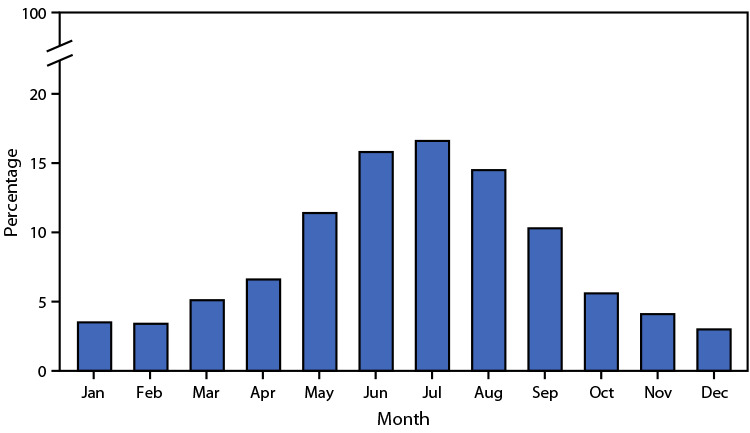
During 2018–2020, 1,508 deaths occurred involving injuries from recreational and nonrecreational use of watercraft. The percentage of deaths each month ranged from 3.0% in December to 16.6% in July. Most deaths (68.6%) occurred during May–September.

**Source:** National Vital Statistics System, Mortality Data. https://www.cdc.gov/nchs/nvss/deaths.htm

